# Reduction of N-Glycolylneuraminic Acid in Human Induced Pluripotent Stem Cells Generated or Cultured under Feeder- and Serum-Free Defined Conditions

**DOI:** 10.1371/journal.pone.0014099

**Published:** 2010-11-23

**Authors:** Yohei Hayashi, Techuan Chan, Masaki Warashina, Masakazu Fukuda, Takashi Ariizumi, Koji Okabayashi, Naoya Takayama, Makoto Otsu, Koji Eto, Miho Kusuda Furue, Tatsuo Michiue, Kiyoshi Ohnuma, Hiromitsu Nakauchi, Makoto Asashima

**Affiliations:** 1 Department of Life Sciences (Biology), Graduate School of Arts and Sciences, The University of Tokyo, Tokyo, Japan; 2 Genome Research Laboratories, Wako Pure Chemical Industries, Ltd., Hyogo, Japan; 3 Department of Biological Sciences, Graduate School of Science, The University of Tokyo, Tokyo, Japan; 4 Division of Stem Cell Therapy, Center for Stem Cell and Regenerative Medicine, Institute of Medical Science, The University of Tokyo, Tokyo, Japan; 5 Laboratory of Cell Cultures, Department of Disease Bioresources, National Institute of Biomedical Innovation, Osaka, Japan; 6 Top Runner Incubation Center for Academia-Industry Fusion, Nagaoka University of Technology, Nagaoka, Japan; 7 Organ Development Research Laboratory, National Institute of Advanced Industrial Science and Technology (AIST), Ibaraki, Japan; Hungarian Academy of Sciences, Hungary

## Abstract

**Background:**

The successful establishment of human induced pluripotent stem cells (hiPSCs) has increased the possible applications of stem cell research in biology and medicine. In particular, hiPSCs are a promising source of cells for regenerative medicine and pharmacology. However, one of the major obstacles to such uses for hiPSCs is the risk of contamination from undefined pathogens in conventional culture conditions that use serum replacement and mouse embryonic fibroblasts as feeder cells.

**Methodology/Principal Findings:**

Here we report a simple method for generating or culturing hiPSCs under feeder- and serum-free defined culture conditions that we developed previously for human embryonic stem cells. The defined culture condition comprises a basal medium with a minimal number of defined components including five highly purified proteins and fibronectin as a substrate. First, hiPSCs, which were generated using Yamanaka's four factors and conventional undefined culture conditions, adapted to the defined culture conditions. These adapted cells retained the property of self renewal as evaluated morphologically, the expression of self-renewal marker proteins, standard growth rates, and pluripotency as evaluated by differentiation into derivatives of all three primary germ layers *in vitro* and *in vivo* (teratoma formation in immunodeficient mice). Moreover, levels of nonhuman N-glycolylneuraminic acid (Neu5Gc), which is a xenoantigenic indicator of pathogen contamination in human iPS cell cultures, were markedly decreased in hiPSCs cultured under the defined conditions. Second, we successfully generated hiPSCs using adult dermal fibroblast under the defined culture conditions from the reprogramming step. For a long therm culture, the generated cells also had the property of self renewal and pluripotency, they carried a normal karyotype, and they were Neu5Gc negative.

**Conclusion/Significance:**

This study suggested that generation or adaption culturing under defined culture conditions can eliminate the risk posed by undefined pathogens. This success in generating hiPSCs using adult fibroblast would be beneficial for clinical application.

## Introduction

Human induced pluripotent cells (hiPSCs) generated by the introduction of defined factors from somatic cells exhibit pluripotency similar to human embryonic stem cells (hESCs) [1], [2]. The broad developmental potential of hiPSCs makes them a possible source of cells for the regenerative medical transplantation of various tissues. However, before hiPSC-derived cells can be used in human transplantation, a number of safety concerns need to be overcome. One such concern is the risk of contamination by undefined pathogens or immunoreactive materials from undefined components used in the culturing of hiPSCs [3]. N-Glycolylneuraminic acid (Neu5Gc) has been identified as an immunoreactive material that contaminates cells in culture. Neu5Gc, a sialic acid found on the cell surface, is considered a xenoantigen for humans because human cells cannot produce Neu5Gc genetically [4], although it can be taken up from the culture environment [5], [6]. Furthermore, most humans have circulating antibodies specific for Neu5Gc. Contamination of hESCs by Neu5Gc was confirmed following culturing under conventional conditions with mouse embryonic fibroblast (MEF)-derived feeder cells and knockout serum replacement (KSR)-supplemented medium [7], [8]. Neu5Gc could therefore be a useful indicator of pathogen contamination in pluripotent stem cell cultures.

Defined culture conditions are therefore required when using hiPSC to avoid contamination from undefined pathogens or immunoreactive materials [7]. KSR-supplemented medium is not defined and thus may contain a variety of contaminating factors [9], [10], [11]. Based on previous findings indicating that the phenotypes of hiPSCs are similar to those of hESCs [1], [2], we hypothesized that hESC culture conditions could also be used for hiPSCs. Previously, we developed a defined serum-free medium, namely hESF9, for culturing hESCs on a type I collagen substrate without feeders [12]. Although several defined culture conditions without feeders for hESCs have been reported, difficulties remain in propagating the undifferentiated hESCs [13], [14], [15], [16]. Recently, we found that adding activin A to hESF9 medium supports robust propagation of hES cells and enhances the stable attachment of these cells to fibronectin [16]. We modified our medium accordingly and subsequently cultured our hESCs on a fibronectin substrate without feeders. The modified medium (hESF9a) comprises a basal medium supplemented with heparin sulphate and five highly purified proteins: bovine pancreatic insulin, human apotransferrin, fatty acid-free bovine serum albumin conjugated with oleic acid, human recombinant fibroblast growth factor (FGF)-2, and human recombinant activin [16].

In the present study, we generated hiPSCs from skin keratinocytes using conventional culture conditions with KSR and feeder cells [17]. The cells were then moved into defined culture conditions in hESF9a medium on fibronectin without feeders. We confirmed that the hiPSCs cultured under defined conditions were pluripotent stem cells on the basis of cell morphology, growth rate, the expression of self-renewal genes, cell differentiation *in vitro*, and teratoma formation *in vivo*. Furthermore, we observed that levels of Neu5Gc decreased steadily in hiPSCs cultured under defined conditions. Finally, we generated hiPSCs from adult dermal fibroblast under defined conditions. For long-term culture, these hiPSCs maintained their pluripotency and normal karyotype.

## Results and Discussion

### Generation of the hiPSC line and adaptation to defined culture conditions

**Figure 1:**
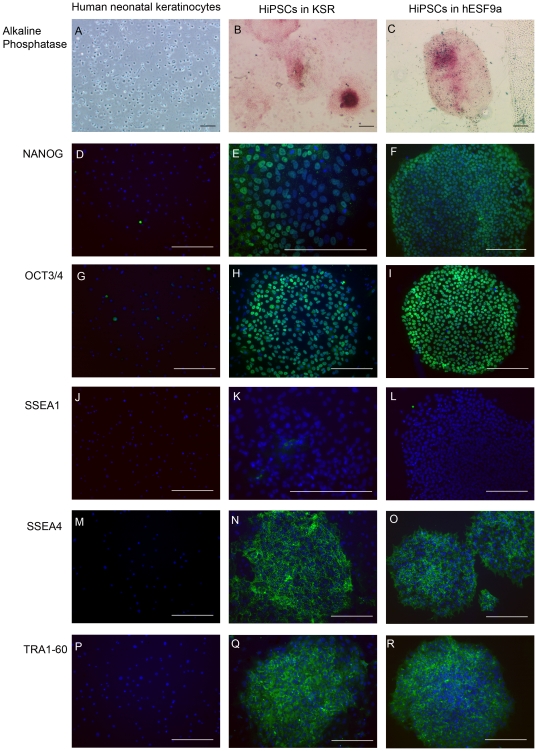
Self-renewal marker expression of pluripotent stem cells in hiPSCs adapted in defined culture conditions. Parental human neonatal keratinocytes (A, D, G, J, M, and P), hiPSC line, UTA1, grown under KSR-based conditions (B, E, H, K, N, and Q), and UTA1 grown under hESF9a-based culture conditions for 5 passages (C, F, I, L, O, and R) were fixed and reacted with antibodies (or stained with alkaline phosphatase substrate, Fast Red). (A–C): Alkaline phosphatase staining. (D–F): Immunocytochemistry of NANOG protein. (G–I): Immunocytochemistry of OCT3/4 protein. (J–L): Immunocytochemistry of SSEA1 antigen. (M–O): Immunocytochemistry of SSEA4 antigen. (P–R): Immunocytochemistry of TRA1-60 antigen. Binding of these antibodies was visualized with AlexaFluor 488-conjugated secondary antibodies (green). Nuclei were stained with DAPI (blue). Scale bars represent 50 µm.

The hiPSC cell line was established from neonatal skin keratinocytes by the infection of amphotropic retroviruses carrying the *OCT4*, *SOX2*, *KLF4*, and C-*MYC* genes using conventional culture conditions with KSR medium and mitomycin C-treated MEF feeder cells (KSR-based conditions) [1], [17], [18]. Under the KSR-based conditions, cells maintained their undifferentiated morphology and expressed markers of pluripotency, namely ALP (Figure 1B), NANOG (Figure 1E), OCT3/4 (Figure 1H), SSEA4 (Figure 1N), and TRA1-60 (Figure 1Q), shown by immunocytochemistry or substrate staining for ALP. However, the cells did not express stage-specific embryonic antigen (SSEA)-1, which is a self-renewal marker of murine ES cells and a differentiation marker of hESCs (Figure 1K). The cell line was designated UTA1.

At passage 18, the UTA1 cells were transferred into the defined culture conditions with hESF9a medium on fibronectin-coated dishes without feeder cells (hESF9a-based conditions) [12], [16]. After a further five passages, the UTA1 cells also expressed the self-renewal markers, ALP (Figure 1C), NANOG (Figure 1F), OCT3/4 (Figure 1I), SSEA4 (Figure 1O), and TRA1-60 (Figure 1R); however, there was no detectable expression of SSEA1 under either culture condition (Figure 1L), suggesting that hiPSCs grown in the hESF9a-based conditions maintained their undifferentiated characteristics. The UTA1 cells steadily proliferated under hESF9a-based conditions for a prolonged culture period, as in the conventional KSR-based conditions (Figure S1). We continued to culture the UTA1 cells in hESF9a-based condition up to 27 passages. These results suggested that the UTA1 cells cultured under the hESF9a-based conditions retained the property of self-renewal.

### In vitro and in vivo differentiation of hiPSCs under defined culture conditions

**Figure 2:**
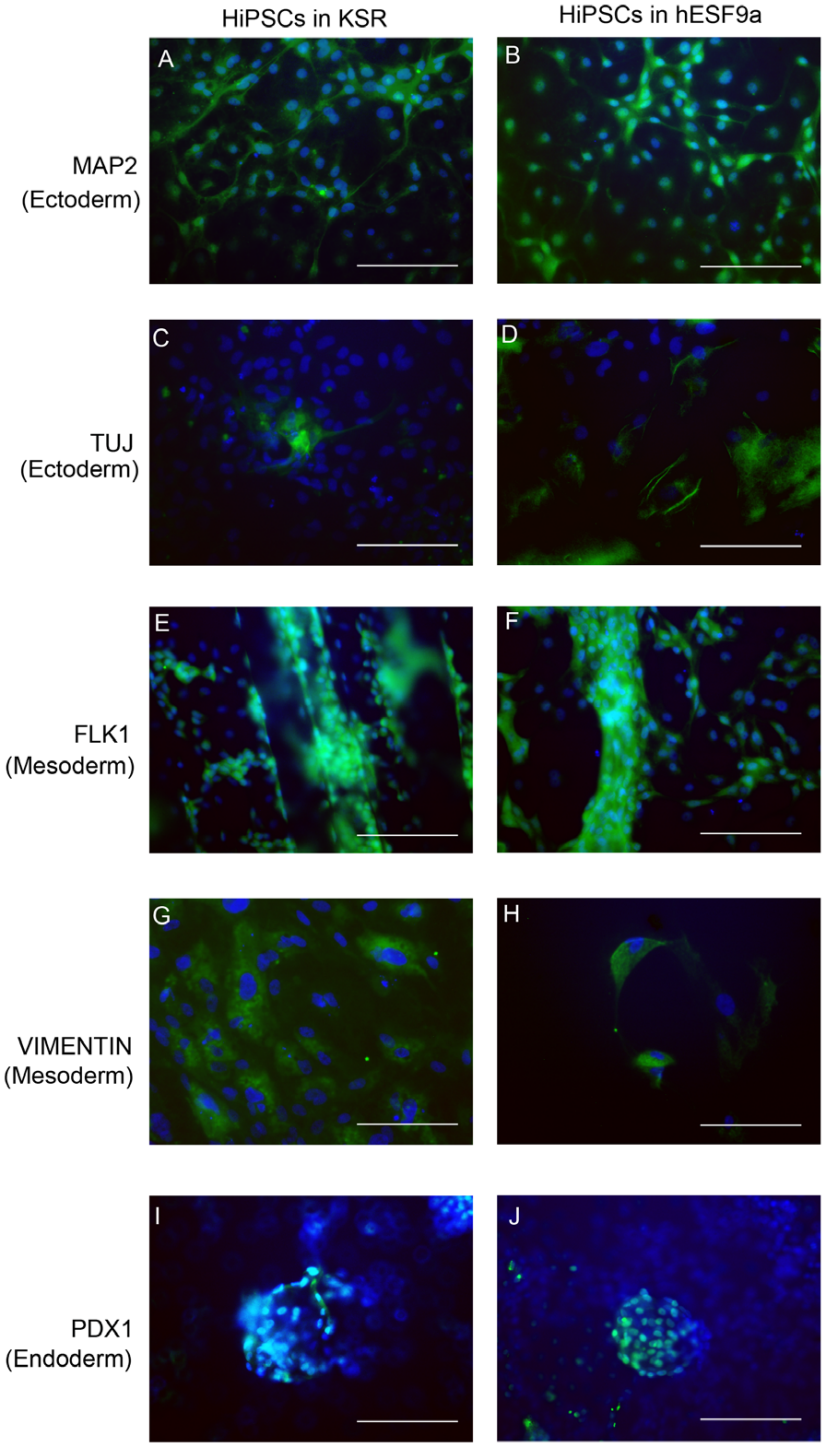
*In vitro* differentiation using embryoid bodies from hiPSCs adapted in defined culture conditions. Immunocytochemistry of MAP2 (A, B), TUJ (C, D), FLK1 (E, F), vimentin (G, H), and PDX1 (I, J) in the differentiated hiPSC line, UTA1, grown under KSR-based conditions (A, C, E, G, I) or hESF9a-based conditions (B, D, F, H, J). Differentiation was performed using embryoid body formation, and the differentiated cells were fixed and reacted with antibodies. Binding of these antibodies was visualized with AlexaFluor 488-conjugated secondary antibodies (green). Nuclei were stained with DAPI (blue). Scale bars represent 50 µm.

**Figure 3:**
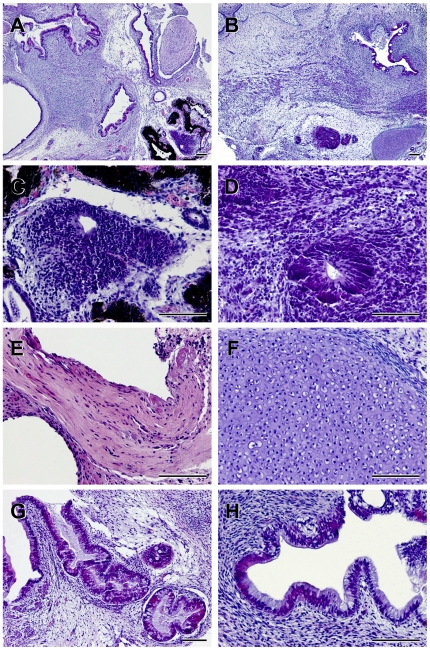
The *in vivo* differentiation using teratoma formation of hiPSCs adapted in defined culture conditions. Teratoma were generated in SCID mice from UTA1 grown under KSR-based and hESF9a-based conditions. Histological analysis with HE staining demonstrated that teratoma formed by the UTA1 cells cultured in both KSR-based (A, C, E, G) and hESF9a-based conditions (B, D, F, H) contained derivatives of all three germ layers. Histology of teratoma derived from UTA1 cultured under KSR-based (A) or hESF9a-based conditions (B). Neural tissues in teratoma derived from UTA1 cultured under KSR-based (C) or hESF9a-based conditions (D). (E): Muscle. (F): Cartilage. (G and H): Intestinal epithelia. Scale bars represent 100 µm.

Differentiation potential in the UTA1 cells grown under KSR- and hESF9a-based conditions was assessed using *in vitro* differentiation assays involving embryoid body generation. After 24 days in differentiation culture conditions, the embryoid bodies contained various types of differentiated cells characterized by germ-layer markers as follows: MAP2 (Figure 2A, B) and TUJ1 (Figure 2C, D) as ectoderm markers; FLK1 (Figure 2E, F) and vimentin (Figure 2G, H) as mesoderm markers; and PDX1 as an endoderm marker (Figure 2I, J). We also validated the pluripotency of UTA1 cells under KSR-based conditions (17 passages) and hESF9a-based conditions (18 passage in KSR-based condition and 12 passage in ESF9a-based condition) by teratoma formation in the testes of severe combined immunodeficient (SCID) mice injected with hiPSCs. Eight weeks after injection, histological analysis demonstrated that the formed teratomas were derived from all three primary germ layers. Neural tissues (ectoderm), muscle (mesoderm), cartilage (mesoderm), and intestinal epithelia (endoderm) were all identified histologically in the hiPSC-derived teratomas (Figure 3). These results suggested that UTA1 cells remained pluripotent to differentiate into all three germ layers when grown under hESF9a-based conditions.

### The level of xenoantigen Neu5Gc in the hiPSC under defined culture conditions

**Figure 4:**
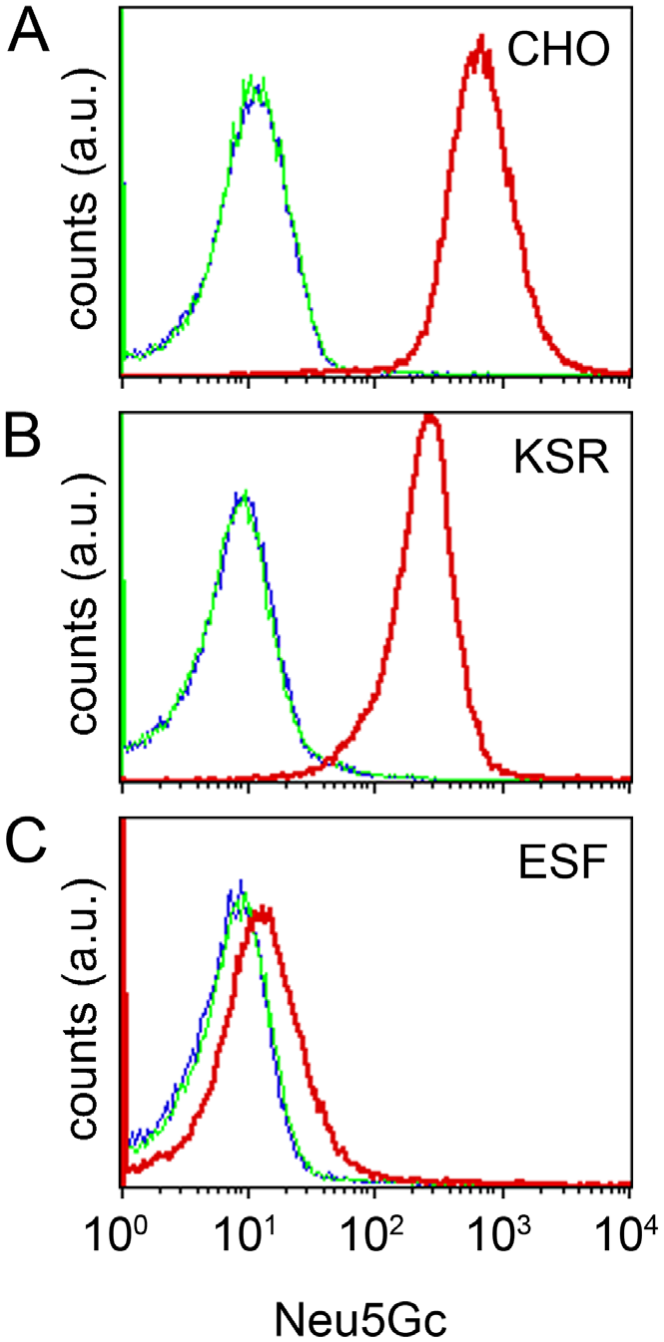
Decreased expression of xenoantigen Neu5Gc in hiPSCs adapted under defined culture conditions. Flow cytometry analysis of Neu5Gc expression. CHO cells were grown in FCS-containing medium (A) and hiPSCs were grown under KSR-based conditions (B) or hESF9a-based conditions (C). The cells were exposed to anti-Neu5Gc antibody (red), control antibody (green), or blocking buffer (blue), and then stained with a secondary antibody for analysis by flow cytometry.

Our data showed that hESF9a culture conditions maintain the pluripotency of hiPSCs. Conventional culture conditions currently use KSR for human ES/iPS cells, and it is accepted that these commercially supplied components may contain undefined animal-derived xenoantigens and pathogens. Because human cells cannot produce Neu5Gc genetically [4], it becomes a useful indicator of xenogenic contamination in human pluripotent stem cells [7]. We therefore examined the expression of Neu5Gc in UTA1 cells grown under KSR- and hESF9a-based conditions by flow cytometry using an antibody against Neu5Gc. The level of Neu5Gc was high in UTA1 cells cultured under the KSR-based conditions (23 passages) and was comparable with that in Chinese hamster ovary (CHO) cells cultured in fetal calf serum (FCS)-containing medium (Figure 4A, B). The Neu5Gc expression decreased almost to negative control levels (with control antibody or no primary antibody) in UTA1 cells cultured under the hESF9a-based conditions (18 passages in KSR-based condition and 27 passages in ESF9a-based condition) (Figure 4C). The conventional culture conditions contain animal-derived components such as MEF, FCS in which the MEFs were cultured, and porcine gelatin. Further, the KSR used for the conventional culture conditions also includes animal-derived components, such as lipid-enriched bovine serum albumin [9], [10], [11]. Hence, the UTA1 cells might metabolically incorporate substantial amounts of Neu5Gc from these factors. However, although the hESF9a-based conditions also contain animal-derived components such as bovine insulin, bovine serum albumin, porcine heparin, and bovine fibronectin, the UTA1 cells incorporate only low amounts of Neu5Gc. These results suggested that culturing of hiPSCs under the defined conditions with purified components could decrease the risk of xenogenic and human-derived pathogens.

### The generation of hiPSC lines using adult dermal fibroblasts under feeder- and serum-free, defined culture conditions from the reprogramming step

**Figure 5:**
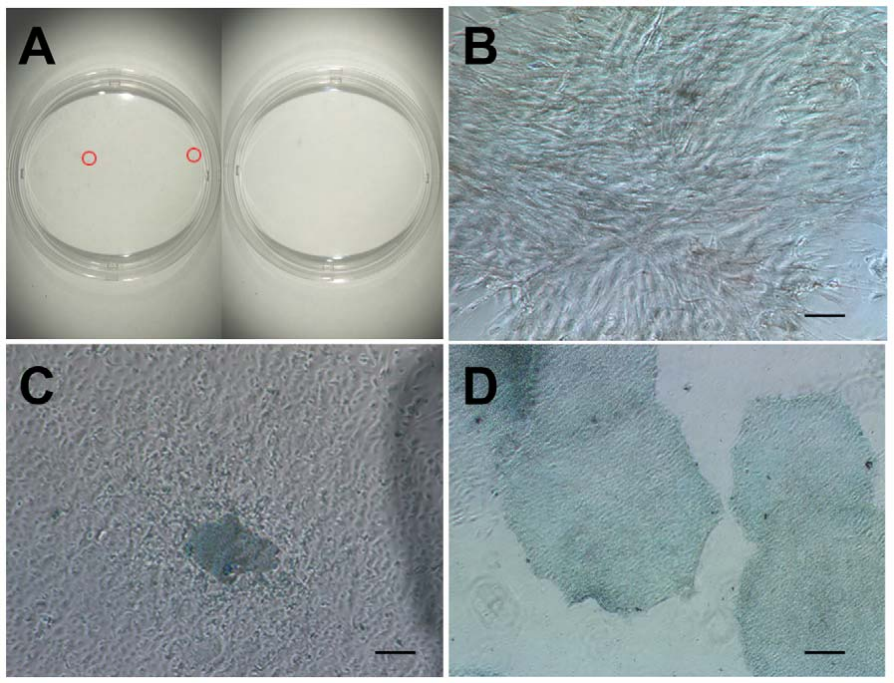
Generation of hiPSC lines under defined culture conditions. (A):ALP staining of HDFs cultured in hESF9a (left) and in TeSR2 (right) at 26 days after virus transduction. Red arrows indicate the ALP-positive hiPSC-like colonies. (B and C): The appearances of transduced HDFs cultured in TeSR2 (B) and in hESF9a (C). Cells were fixed and stained with ALP substrate BM purple at 26 days after virus transduction. (D): ALP staining of the hiPSC-like colony picked up and cultured under hESF9a conditions at five passages. Scale bars represent 50 µm.

**Figure 6:**
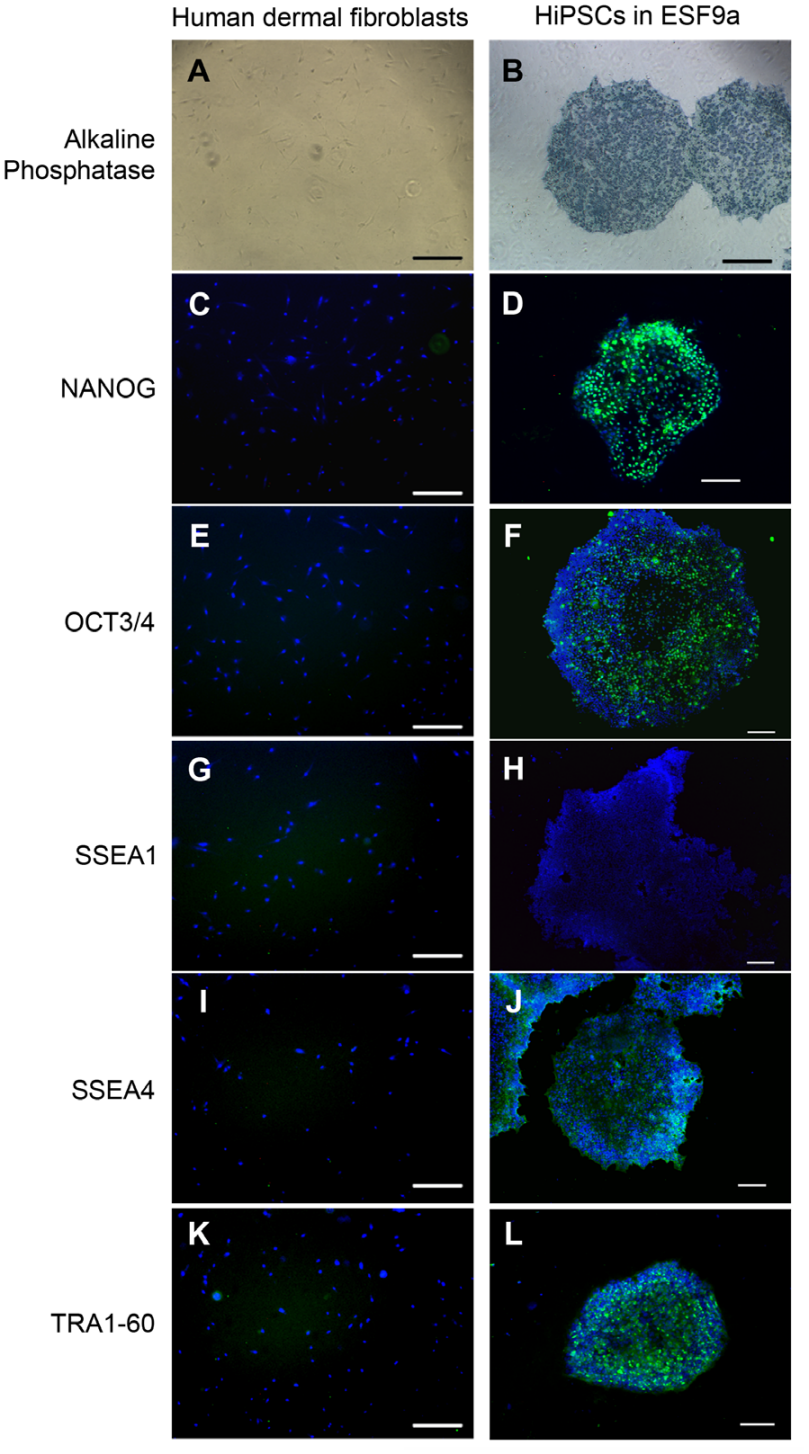
Self-renewal marker expression of pluripotent stem cells in hiPSCs generated and maintained in defined culture conditions. Parental human dermal fibroblasts (A, C, E, G, I, and K), and hiPSC line, SF2-2 generated and maintained under the ESF9-based conditions (B, D, F, H, J, and L) were fixed and reacted with antibodies (or stained with alkaline phosphatase substrate, BM Purple). (A and B): Alkaline phosphatase staining. (C and D): Immunocytochemistry of NANOG protein. (E and F): Immunocytochemistry of OCT3/4 protein. (G and H): Immunocytochemistry of SSEA1 antigen. (I and J): Immunocytochemistry of SSEA4 antigen. (K and L): Immunocytochemistry of TRA1-60 antigen. Binding of these antibodies was visualized with Alexafluor 488-conjugated secondary antibodies (green). Nuclei were stained with DAPI (blue). Scale bars are 50 µm.

**Figure 7:**
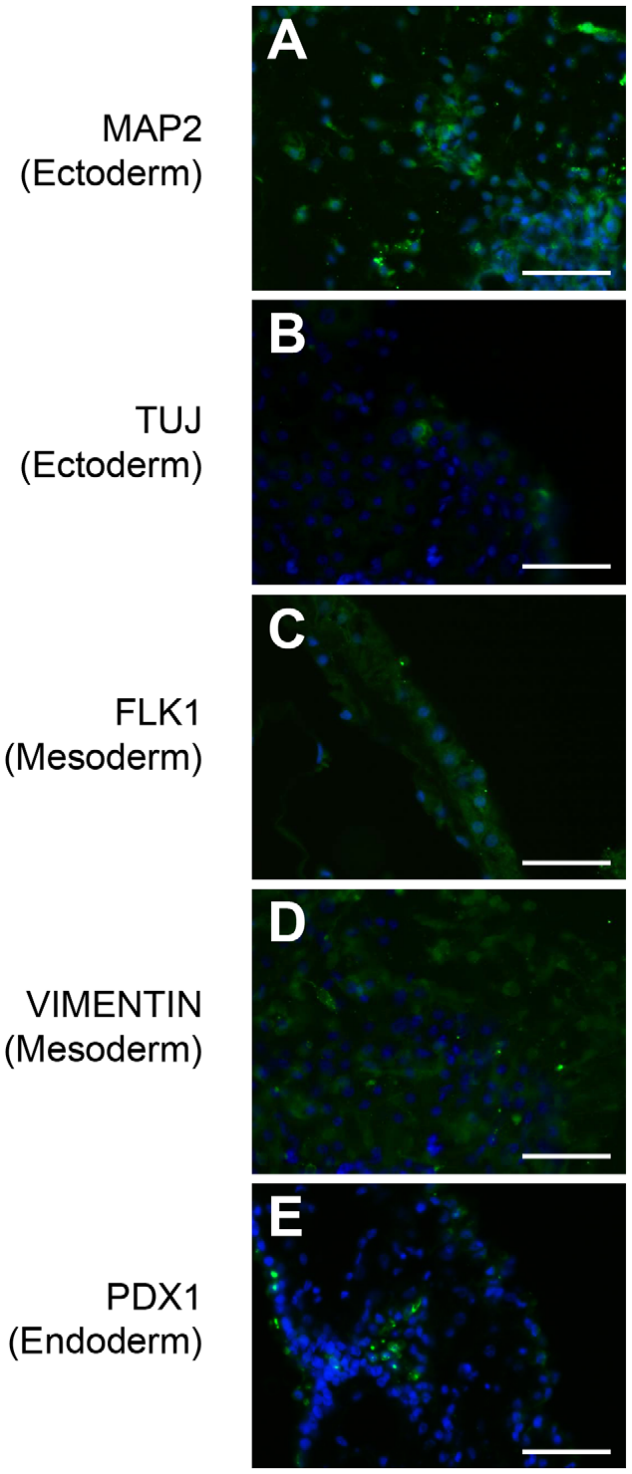
*In vitro* differentiation using embryoid bodies from hiPSCs generated and maintained in defined culture conditions. Immunohistochemistry of MAP2 (A), TUJ (B), FLK1 (C), VIMENTIN (D), and PDX1 (E) in the differentiated hiPSC line, UTA-SF-2-2, grown under hESF9a-based conditions. Differentiation was performed using embryoid body formation. The tissues in embryoid bodies were fixed and reacted with antibodies. Binding of these antibodies was visualized with AlexaFluor 488-conjugated secondary antibodies (green). Nuclei were stained with DAPI (blue). Scale bars are 50 µm.

**Figure 8:**
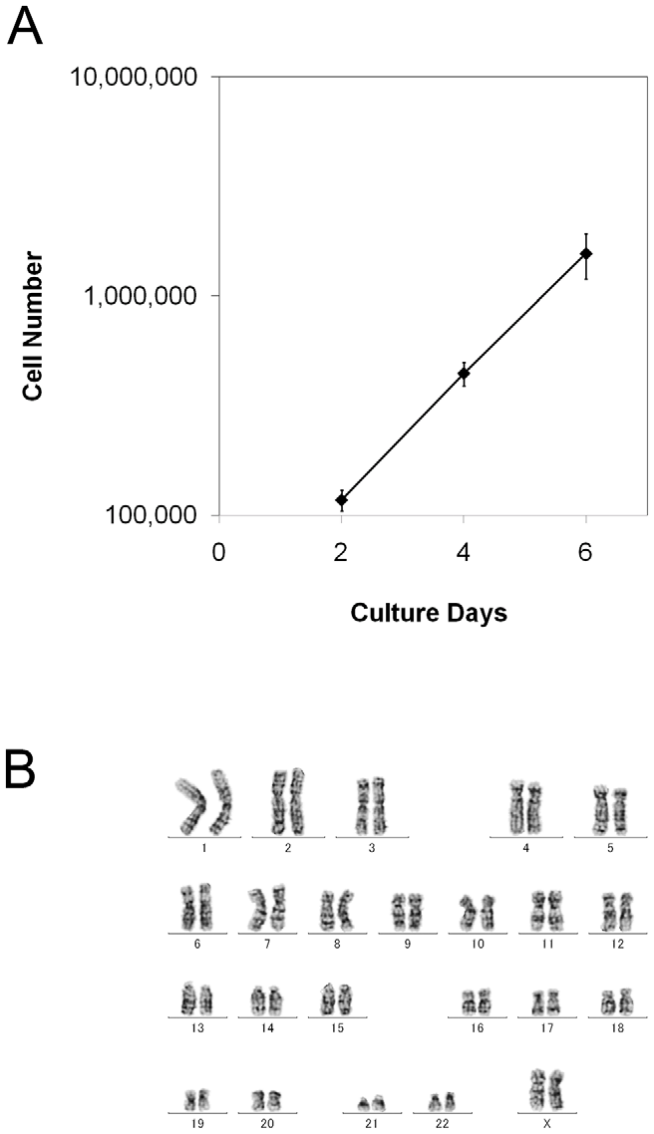
Cell growth and karyotype of hiPSCs generated and maintained in defined culture conditions. (A): Growth curves for the hiPSC line. UTA-SF2-2 hiPSCs cultured under hESF9a-based conditions at passages 29, 30, and 31 were seeded in a 6-well plate coated with fibronectin and counted every 48 h. The values are the mean ± SEM (n = 3). (B): Representative G-banded karyotype of chromosome in a UTASF2-2 hiPSC at passage 31 maintained under hESF91-based conditions.

**Figure 9:**
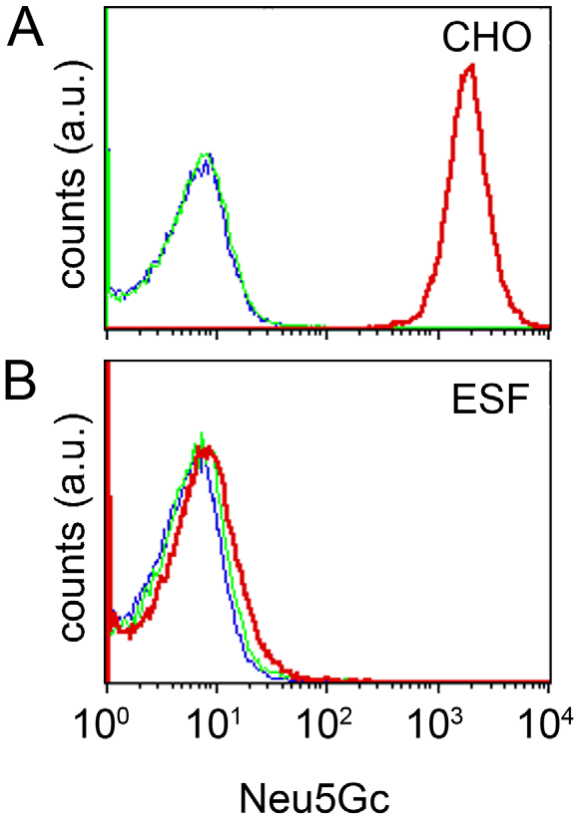
Flow cytometry analysis of xenoantigen Neu5Gc expression in hiPSCs generated and maintained in defined culture conditions. CHO cells grown in FCS-containing medium (A) and UTA-SF2-2 hiPSCs established under hESF9a-based conditions (B) were exposed to anti-Neu5Gc (red) or control antibody (green), or blocking buffer (blue). Then stained with a secondary antibody for analysis by flow cytometry.

Next, we examined whether hiPSCs were generated from the reprogramming step under our feeder-free, defined culture conditions. For comparison, we used another defined xeno-free TeSR2 media, which was modified from mTeSR1 medium [13], for culturing human pluripotent stem cells. Adult human dermal fibroblasts (HDF) were infected with amphotropic retroviruses carrying the *OCT4*, *SOX2*, *KLF4*, and C-*MYC* genes and were cultured under hESF9a-based conditions or under TeSR2-based conditions (with TeSR2 medium on matrigel-coated dishes). After 26 days of culture, we detected hiPSC-like colonies by staining for ALP substrate or by their morphologies (Figure 5A). While no hiPSC-like colonies were detected under the TeSR2-based conditions, eight hiPSC-like colonies were detected under the hESF9a-based condition. We confirmed these results by independent multiple experiments (0 ALP-positive colonies/2 experiments in TeSR2-based conditions and 3 ALP-positive colonies/2 experiments in hESF9a-based conditions) (Figure 5B and C). In another experiment of the hiPSCs induction under hESF9a-based conditions, we picked up hiPS-like colonies for expansion under the same culture conditions. The ALP activity was maintained at 5 passages (Figure 5D). After 25–26 passages, we confirmed self-renewal marker expression and differentiation potential in these cell lines by immunocytochemistry (Figure 6, 7). One of the cell lines, designated UTA-SF2-2, retained proper proliferation rates for human pluripotent stem cells (Figure 8A; population doubling time: 28.2±4.9 h). Karyotype analysis revealed that UTA-SF2-2 cells at passage 31 was 46XX (Figure 8B). Finally, we examined Neu5Gc expression in the UTASF2-2 line by flow cytometry and showed that the levels of Neu5Gc in passage-30 cells were almost negative, as in negative control cells (Figure 9). Our established cell lines therefore showed little or no Neu5Gc contamination, suggesting that the hESF9a-based culture conditions generated hiPSCs steadily from reprogramming step using adult HDF and supported their pluripotency for long-term culture with less contamination of pathogens.

Recently, the generation of induced pluripotent stem cells under xeno-free culture conditions has been reported [19], [20]. These culture conditions contain human plasma or xeno-free KSR on irradiated human fibroblasts. However, the components of KSR or xeno-free KSR are not publicly available. For clinical application, all the components used should be traceable and also widely reviewed. Our defined culture methods without feeder cells make it easier to track all the components because the minimum essential components, only five highly purified proteins with heparin, are added into the basal medium. Previously, human iPSCs were also generated using a defined medium, mTeSR1 from not adult but neonatal HDF (ADA) [21] or adult adipose stem cells [22]. Sun *et al*. also reported that no iPS-like colonies were generated from neonatal HDF (IMR90) using mTeSR1 medium [22]. Together with our results that hiPSCs from adult HDF were derived under defined culture conditions for the first time and steadily, our hESF9a medium is suitable to generate hiPSCs from adult HDF. Compared with our hESF9a medium, mTeSR1 and TeSR2 medium contain a variety of chemicals (inorganic chemicals, trace minerals, lipids, surfactants, and amino acid derivatives) that regulate signal transduction and metabolism [13]. Although these chemicals may enhance the self-renewal of human pluripotent stem cells, some may inhibit the reprogramming steps during human iPSC generation.

### Conclusions

This study demonstrated that defined culture conditions that were developed for hESCs are also applicable for hiPSCs. The hiPSCs were generated under conventional KSR-based conditions and then adapted to the defined hESF9a-based condition. Moreover, we succeeded to generate the hiPSCs from adult HDF under hESF9a-based conditions. The levels of xenoantigen Neu5Gc were markedly decreased in the hiPSCs which were either adapted or generated in the hESF9a-based condition. The hESF9a media consists of a basal medium and known components, reducing the risk of contamination from undefined pathogens and antigens. Taken together, our findings suggested that the defined culture conditions described herein are suitable for culturing hiPSCs with the added benefit of eliminating contamination risks caused by undefined factors. The defined culture condition provides a safer source of hiPSCs for potential clinical applications.

## Materials and Methods

### hiPSCs induction and cell culture

Human neonatal keratinocytes were purchased from Invitrogen (Carlsbad, CA). An hiPS cell line was generated using the VSV-G-pseudotyped retroviral vector system carrying *OCT4*, *SOX2*, *KLF4*, and *C-MYC* as described previously [18]; the line was designated UTA1. UTA1 cells were maintained in DMEM-F12 medium (Invitrogen) supplemented with 20% KSR (Invitrogen), 0.1 mM 2-mercaptoethanol (Sigma, St. Louis, MO), MEM non-essential amino acids (Invitrogen), and 5–10 ng/mL recombinant human basic FGF (Peprotech, Rocky Hill, NJ) on mitomycin C-treated mouse embryo fibroblast feeder cells. For subculturing, the cells were detached from the culture dish using CTK medium [17]. The mouse embryonic fibroblasts were cultured in fibroblast medium (DMEM medium supplemented with 10% fetal calf serum, 1% penicillin, and 1% streptomycin) on gelatin-coated dishes.

UTA1 cells at passage number 18 were transferred into a defined feeder- and serum-free culture condition, the hESF9a-based condition (with hESF9a medium and fibronectin coat), and passaged a further 5 times at least before assaying. The hESF9a medium comprises hESF-Grow medium (Cell Science & Technology Institute, Miyagi, Japan) supplemented with 10 µg/mL of bovine pancreas insulin (Sigma I-5500), 5 µg/mL human apotransferrin (Sigma T-1147), 10 µM 2-mercaptoethanol (Sigma M-7522), 10 µM ethanolamine (Sigma E-0135), 20 nM sodium selenite (Sigma S-9133), 4.7 µg/mL of oleic acid conjugated with 0.5 mg/mL of fraction V fatty acid-free bovine serum albumin (Sigma O-3008), 100 ng/mL L-ascorbic acid-2-phosphate (Wako, Osaka, Japan, 013-196411), 100 ng/mL heparin sodium salt from porcine intestinal mucosa (Sigma H-3149), 10 ng/mL human recombinant fibroblast growth factor 2 (FGF-2, Peprotech 100-18B), and 10 ng/mL human recombinant activin A (Ajinomoto Pharmaceuticals, Japan) [12], [16]. The culture dishes were coated with 2 µg/cm^2^ fibronectin from bovine plasma (Sigma F-1141) in PBS for at least 30 min at 37°C, and then excess solution was removed. For subculturing, the cells were detached from the culture dish using 50∼300 µg/mL dispase (Invitrogen 17105-041) in hESF9a medium and replated in hESF9a medium with 5 µM ROCK inhibitor (Y-27632; Wako Pure Chemical Industries, Ltd., Japan). Medium changes were made every day with hESF9a medium.

Chinese hamster ovary (CHO) cells (No. 85050302, European Collection of Cell Culture) were maintained in DMEM-F12 (Invitrogen) supplemented with 10% fetal calf serum, 1% penicillin, and 1% streptomycin.

### Generation and maintenance of hiPSCs in feeder- and serum-free defined culture condition from reprogramming step

Four hiPSC lines, UTA-SF2-1, UTA-SF2-2, UTA-SF3-1, and UTA-SF3-2, were generated from primary adult human dermal fibroblasts (female of 41, 46, or 51 yr old; 106-05a, cell applications, inc. San Diego, CA) under hESF9a-based conditions. The HDFs were seeded at 8×10^5^ cells/dish in 10-cm petri dishes coated with gelatin in the fibroblast medium. The VSV-G-pseudotyped retroviral vector system carrying OCT4, SOX2, KLF4, and C-MYC was added to HDF cultures. After two 24-hour-exposures of virus, the transduced HDFs were replated at 6×10^4^ cells/dish onto 10-cm petri dishes coated with fibronectin or matrigel in the fibroblast medium. On the next day, the medium was changed to hESF9a (on fibronectin-coated dishes) or TeSR2 (on matrigel-coated dishes), and thereafter changed daily. At around 30 days after transduction, hiPSC-like colonies were picked up and cultured in the same defined culture conditions as described for the hiPSC UTA1 cell lines.

### Alkaline phosphatase (ALP) staining

Cells were fixed with 4% paraformaldehyde (PFA) in phosphate-buffered saline (PBS) at room temperature for 10 minutes. The fixed cells were washed with PBS three times and then twice with alkaline phosphatase (ALP) buffer. Finally, these samples were stained with ALP substrate Fast-Red (Nichirei, Japan) or BM purple (Roche, Switzerland) for 30 minutes at room temperature.

### Embryoid body formation

*In vitro* differentiation was induced by the formation of embryoid bodies as described previously [12]. Briefly, undifferentiated hiPSCs were cultured in DMEM with 10% FCS for 8 days in low-attachment plates (Corning, Corning, NY). Then, floating embryoid bodies were replated onto gelatin-coated dishes in the same culture medium for 16 days.

### Immunocytochemistry

Immunocytochemistry was performed as described previously [23], [24], [25]. Briefly, hiPSCs were fixed in 4% PFA, permeabilized with 0.1% Triton X-100, blocked with 1% BSA, and then reacted with primary antibodies. The primary antibody binding was visualized with AlexaFluor 488-conjugated anti-rabbit, anti-mouse, and anti-goat IgG or AlexaFluor 594-conjugated donkey anti-mouse, anti-rabbit, or anti-goat IgG (Invitrogen). The following primary antibodies were used: anti-FLK1 antibody (Chemicon, Billerica, MA; 1∶100), anti-MAP2 antibody (Chemicon; 1∶200), anti-PDX1 antibody (Chemicon; 1∶100), anti-NANOG antibody (Reprocell, Tokyo, Japan; 1∶200), anti-OCT3/4 antibody (Santa Cruz Biotechnology, Santa Cruz, CA 1∶100), anti-SSEA1 antibody (Kyowa, Tokyo, Japan; 1∶100), anti-SSEA4 antibody (eBiosciences, San Diego, CA; 1∶100), anti-TUJ antibody (Chemicon; 1∶100), and anti-vimentin antibody (Santa Cruz Biotechnology; 1∶200).

### Teratoma formation assay

For teratoma formation assays, approximately 3 million hiPSCs were suspended in 60 µL of PBS and injected into the testes of anesthetized severe combined immunodeficient (SCID) mice. The tumors were excised 8 weeks after injection, fixed in 4% PFA, embedded in paraffin, and then sectioned at 8 µm. The histology of formed teratomas was analyzed using hematoxylin-eosin (HE) staining. The Institutional Animal Care and Use Committee of the Institute of Medical Science, University of Tokyo approved the use of experimental animals (the permit number was PA09-4).

### Flow cytometry

Flow cytometry for Neu5Gc was performed as described previously [7]. Briefly, all cells were removed from culture dishes using 0.02% (w/v) EDTA-4Na in PBS. The hiPSCs cultured under KSR-based conditions were then replated on plastic dishes and incubated for 1 hour to remove the MEFs, and then 0.5–2.5×10^6^ cells were incubated with a chicken anti-Neu5Gc (Gc-Free, 1∶100 dilution) antibody or a control antibody (Gc-Free, 1∶100 dilution) in PBS containing blocking buffer (Gc-Free), but without Neu5Gc. The cells were finally incubated with a donkey anti-chicken IgY secondary antibody conjugated to Cy5 (Jackson; diluted 1∶500 in PBS containing blocking buffer). A FACS-Calibur (BD) was used for data acquisition.

## Supporting Information

Figure S1 Cell growth of hiPSCs cultured under defined culture conditions. Growth curves for the hiPSC line, UTA1, cultured under KSR-based or hESF9a-based conditions. Growth curves were calculated from each passage split ratio. The relative cell number was set as 1 when the hiPSCs were cultured at passage 18 for conventional feeder conditions or at passage 5 for defined culture conditions.
(2.10 MB TIF)
