# Supplementary figures and images for: Reduction of N-Glycolylneuraminic Acid in Human Induced Pluripotent Stem Cells Generated or Cultured under Feeder- and Serum-Free Defined Conditions
Source: PLoS One. 2010 Nov 23;5(11):e14099. doi: 10.1371/journal.pone.0014099 (PMC2990711; doi:10.1371/journal.pone.0014099)

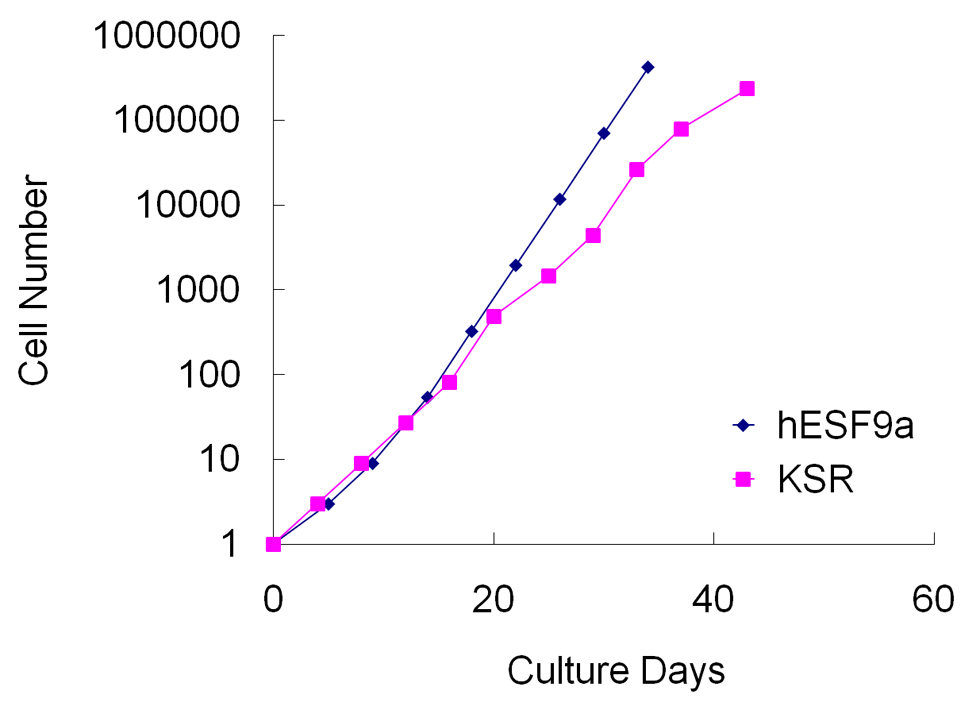

Supplement: Figure S1 — Cell growth of hiPSCs cultured under defined culture conditions. Growth curves for the hiPSC line, UTA1, cultured under KSR-based or hESF9a-based conditions. Growth curves were calculated from each passage split ratio. The relative cell number was set as 1 when the hiPSCs were cultured at passage 18 for conventional feeder conditions or at passage 5 for defined culture conditions. (2.10 MB TIF) [file pone.0014099.s001.tif]
